# The Implicit Aesthetic Preference for Mobile Marketing Interface Layout—An ERP Study

**DOI:** 10.3389/fnhum.2021.728895

**Published:** 2021-09-30

**Authors:** Shu Wang, Chonghuan Xu, Liang Xiao, Austin Shijun Ding

**Affiliations:** ^1^School of Management and E-Business, Zhejiang Gongshang University, Hangzhou, China; ^2^Modern Business Research Center, Zhejiang Gongshang University, Hangzhou, China; ^3^School of Business Administration, Zhejiang Gongshang University, Hangzhou, China; ^4^Institute of Applied Psychology, School of Business Administration, Zhejiang Gongshang University, Hangzhou, China; ^5^Sobey School of Business, Saint Mary's University, Halifax, NS, Canada

**Keywords:** neuroaesthetics, cognitive neuroscience, aesthetic preference, interface layout, neuromarketing, ERPs, P2, LPP

## Abstract

Businesses and scholars have been trying to improve marketing effect by optimizing mobile marketing interfaces aesthetically as users browse freely and aimlessly through mobile marketing interfaces. Although the layout is an important design factor that affects interface aesthetics, whether it can trigger customer's aesthetic preferences in mobile marketing remains unexplored. To address this issue, we employ an empirical methodology of event-related potentials (EPR) in this study from the perspective of cognitive neuroscience and psychology. Subjects are presented with a series of mobile marketing interface images of different layouts with identical marketing content. Their EEG waves were recorded as they were required to distinguish a target stimulus from the others. After the experiment, each of the subjects chose five stimuli interfaces they like and five they dislike. By analyzing the ERP data derived from the EEG data and the behavioral data, we find significant differences between the disliked interfaces and the other interfaces in the ERP component of P2 from the frontal-central area in the 200–400 ms post-stimulus onset time window and LPP from both the frontal-central and parietal-occipital area in the 400–600 ms time window. The results support the hypothesis that humans do make rapid implicit aesthetic preferences for interface layouts and suggest that even under a free browsing context like the mobile marketing context, interface layouts that raise high emotional arousal can still attract more user attention and induce users' implicit aesthetic preference.

## 1. Introduction

The global pandemic of COVID-19 has created a huge shift for both markers and customers by introducing a heavier dependency on mobile marketing (Ayush et al., 2020). The development of mobile marketing during the COVID-19 pandemic has witnessed a drastic growth of homogeneous marketing contents that are of little value to the users delivered through mobile marketing. The rapid aimless browsing pattern of users navigating through these contents is identified as free-browsing (Liu et al., 2019). The interface designs that carry mobile marketing contents attract user interaction intentions under this browsing paradigm before a careful scrutiny of the marketing contents. To interact with the mobile marketing system is not only to obtain information and use functionalities, but also to observe and experience the aesthetics of the interface layout. Studies have suggested that everyday visual stimuli such as geometric graphs can trigger human implicit aesthetic preferences without explicit appraisal and decision-making (Handy et al., 2010). However, under the free-browsing paradigm, the mechanism of how the interface layout affects the human aesthetic experience is still not well-understood, and whether the interface layout as a significant interface design feature can cause human implicit aesthetic preference or not remains unexplored.

Researchers and designers have made a lot of efforts including guidelines and principles (Blair-Early and Zender, 2008) to make interfaces more attractive to users (Sears and Jacko, 2009) by improving the interaction functionality and usability of interfaces (Goodwin, 1987). However, management and marketing studies suggest that the affecting factors of design (Edell and Burke, 1987) can shape the emotions of users (Darden and Babin, 1994) and that the visual aesthetics related to feelings and emotions have substantial effects on users' perception of interface functionality and usability (Tractinsky et al., 2000). Stemming from psychology, the studies of interface design from the visual aesthetics perspective utilize users' aesthetic preferences to study the attractiveness of interface designs. Aesthetic preference is an important part of user preference that influences marketing effects (Sevilla and Townsend, 2016). A series of interrelated theories have been developed while studying the causes and the internal logic of users' visual aesthetic preferences. Among them are the classic aesthetic preference theory (Bornstein and Berlyne, 1974), prototype preference theory (Martindale, 1981), processing fluency theory (Reber et al., 2004), and many other theories derived from the arousal dynamics theory proposed by psychobiology (Lavie and Tractinsky, 2004). They provide a solid basis for experimental aesthetics and offer explanations for users' aesthetic preferences (Mõttus et al., 2016, 2017; Bhandari et al., 2019). However, these traditional theories from psychology and experimental aesthetics can neither explain the formation of individual aesthetic experience nor provide effective evidence for the physiological basis of aesthetic preferences.

In recent years, neuroaesthetics, which has emerged from cognitive neuroscience (Skov and Vartanian, 2009), has provided theoretical basis and methodology by offering physiological evidence for user aesthetic preference studies. The science of art theory provides the theoretical basis for explaining the neurological mechanism of aesthetic experience (Ramachandran and Hirstein, 1999). The cognitive appraisal theory of emotion suggests that the processing of stimuli can trigger aesthetic-related emotional responses and provides a theoretical basis for studying the aesthetic preferences of interfaces from cognitive neuroscience (Silvia, 2005). Studies that combine multiple interface design features (such as color, font, shape, etc.) are usually carried out by quantifying design aesthetics. They are usually directed to the result that multiple interface design features influence users' aesthetic preferences (Moshagen and Thielsch, 2010; Mttus et al., 2013) making it hard to extract further implications from the intertwined conclusions. Therefore, research from a single interface design feature (Laarni et al., 2005; Sheedy et al., 2005) is more promising and meaningful, both theoretically and practically.

The layout is a vital interface design feature. It is the reasonable arrangement of interface visual elements according to certain objective constraints, so as to ensure smooth communication between humans and machines (Deng and Wang, 2020). The general practice of measuring the aesthetics of interface layout is quantitative evaluation. From the aesthetic quantification perspective, interface layout can be expressed by a set of attributes (Ngo et al., 2003). The attributes of balance (Streveler and Wasserman, 1984), “overall density,” “local density,” “combination,” “complexity” (Tullis, 1983), “symmetry” (Balinsky, 2006), “cohesion” (Constantine, 1996), “order,” and “simplicity” (Deng and Poole, 2010), etc. have all been used for on-screen interface layout aesthetic evaluation. Some studies decompose interface layout into a set of attributes and study the influence of the attributes on layout aesthetics. Salimun C. decomposed the interface layout into six aesthetic-related attributes and ranked the attributes according to their influences on user preference (Salimun et al., 2010). Altaboli decomposed interface layout into three attributes and studied the influences of these attributes on aesthetic perception (Altaboli and Lin, 2011). Other studies applied the attributes to study the aesthetic evaluation of interface layout. Coleman adopted an aesthetic evaluation method based on aesthetic optimization when studying the layout of self-adjust graphs (Coleman and Parker, 1996). Purchase adopted an experimental evaluation method of aesthetic threshold to study the layout of UML graph interface (Purchase et al., 2002). Harrington used a heuristics approach to measure the aesthetics of automated document interface layout (Harrington et al., 2004).

Aside from the quantitative methods, the cognitive appraisal theory of emotion and neuroaesthetics emphasizes human processing of visual stimuli while the latter goes a step further by offering the physiological evidence of this processing. Traditional research on visual aesthetics of interface layout from the neuroscience perspective mainly focuses on supporting the improvement of interface layouts. Guo studied multiple interface design elements, including layout, addressing the problem of optimizing game navigation interfaces with the help of event-related potentials (ERP) methodology. They have proposed suggestions to improve the user experience of game navigation interface designs by exploiting the correlation of the ERP components of P2 and N2 with perceived experience and aesthetic appraisal (Guo et al., 2019). Other neuroaesthetics studies have revealed that visual stimuli such as logos, images, geometric figures, etc. can all trigger people's implicit aesthetic preferences. This phenomenon does not require the interference of decision-making (Bargh and Ferguson, 2000; Handy et al., 2010). However, as a substantial feature of interface design, whether layouts can evoke people's implicit aesthetic preferences still lacks sufficient scientific evidence.

This work studies the relationship between interface layouts and human aesthetic preference on mobile marketing interface with the neuromarketing methodology of ERP (event-related potentials). The hypothesis is that interface layout can evoke people's rapid implicit aesthetic preference under the free-browsing paradigm in mobile marketing. Aesthetic preference is a particular cognitive process that processes visual stimuli in a hierarchical order and can ultimately reflect some specific forms of neuro activities (Cela-Conde et al., 2013; Vartanian and Skov, 2014; Menzel et al., 2018). The ERP methodology is frequently used in consumer neuroscience, neuromarketing, and neuroaesthetics studies (Sánchez-Núñez et al., 2021). By recording brainwave (electroencephalogram, EEG) signals on the scalp and analyzing the tiny changes of potentials in brainwaves evoked by internal or external events (Handy, 2005), the ERP methodology is a non-invasive technology with the outstanding advantage of high temporal resolution. This makes ERP a perfect tool to analyze the rapid cognitive response evoked by the stimulus. The ERP components have shown remarkable reliability in reflecting the cognitive process of processing the stimulus. Studies have found that ERP components, including P300, EPN, LPP, etc., can all be evoked by emotional stimuli. The evocation can occur even under free-browsing on stimulus materials without explicit appraisal activities of the participants (Rozenkrants and Polich, 2008; Hajcak et al., 2010; Leite et al., 2012). The first ERP experiment related to human aesthetic appraisal was implemented in 2000 by Jacobsen et al. (Jacobsen and Höfel, 2001). Their study showed that a negative ERP wave could be detected in the time window of 300–400 ms from the frontal lobe after the onset of the stimulus. This ERP wave is more pronounced for non-aesthetic geometric figures than aesthetic ones. Since then, ERP methodology has grown its popularity in researching aesthetic preference evoked by stimulus (Deng and Poole, 2010; Zhang et al., 2011; Li et al., 2015; Lü et al., 2016). By studying the related literature and methods, we designed an ERP experiment to study the aesthetic preference for interface layout in this work.

In the experiment, all subjects are presented with a series of visual stimuli in the form of images. These stimuli are a series of mobile marketing advertisement interfaces and a special figure as the target stimulus. The subjects' ERP brainwaves are simultaneously recorded as they were watching the stimuli. For the stimuli, colors are removed by graying out the images. The content, font, and format are all kept identical, with the only difference in their layouts. Multiple trial groups are arranged in the experiment, and the target stimulus appears 10 times in each trial group. Subjects are asked to give feedback by pressing a button as soon as the target stimulus shows up. This response is the only task they are instructed to finish through the whole experiment. After finishing all the trial groups, subjects are asked to choose five interfaces they like most and five they dislike most. The choices of liked/disliked interfaces and the spontaneous visual aesthetic stimulus process in the brain nerve structure reflect the subjects' explicit and implicit preferences for the interfaces, respectively. The average ERP waveforms evoked by the liked, disliked, and other (interfaces other than the liked and disliked) interfaces, are derived from four post-stimulus onset time windows: 150–200, 200–300, 300–400, and 400–600 ms. ERP components in the first three time windows are closely connected to the perception and classification of visual stimuli as well as the early cognitive process of aesthetic preference (Höfel and Jacobsen, 2007; de Tommaso et al., 2008; Müller et al., 2010; Wang et al., 2012), while the ERP component of LPP in the last time window is related to deep aesthetic preference (Hajcak et al., 2009; Handy et al., 2010).

To address our hypothesis, if humans make rapid implicit aesthetic preferences for interface layouts under the free-browsing paradigm in mobile marketing, the stimuli (interfaces liked or disliked by subjects) will evoke multiple ERP components sensitive to aesthetic preference and affect brainwaves in various time windows after stimuli onsets. By analyzing the ERP components, we can find the physiological evidence of whether interface layouts can evoke human rapid implicit aesthetic preference. The findings of our study are critical to understand the mechanism of how the interface layout affects the human aesthetic experience and can benefit the mobile marketers by providing a deeper understanding of their marketing interface designs, especially during the COVID-19 pandemic.

## 2. Materials and Methods

### 2.1. Subjects

Learnt from the studies of Ma et al. which recruited 17 subjects to study the implicit aesthetic experience of architecture using ERP (Ma et al., 2015) and Li et al. which recruited 17 subjects to study the aesthetic preference of Chinese typefaces using ERP (Li et al., 2015), we recruited 20 subjects from local university students and staff, 10 men and 10 women aged between 20 and 35 (Mean = 24.3, SD = 3.8), to carry out our ERP experiment. One of the subjects forgot to take off his smart watch during the experiment. Thus, his data were eliminated from the study. At last, data of 19 subjects were included in the study. All subjects had normal or correct to normal vision. No other eye or visual impairment symptoms, no mental or psychological illness, no alcohol or drug intake, no smoking were detected before the experiment, and they had adequate sleep in the past 24 h. None of the subjects had received education or training in fine art, aesthetics, or design. Meanwhile, to ensure the familiarity of the marketing content in the experimental materials, all the subjects were subscribers of the same ISP (China Mobile). The subjects all signed an informed consent form and got paid after they had finished the experiment.

### 2.2. Materials

When Jacobsen et al. used ERP technology to study the relationship between symmetry and aesthetic appraisal, they made abstractions and designed the materials into simple geometric figures (Jacobsen and Höfel, 2001). Guo et al. created their materials into abstract frameworks and used ERP technology to study the aesthetic appraisal of smartphone designs (Guo et al., 2016). Unlike their approaches, to simulate the real scene of encountering a specific visual stimulus in a free-browsing context, we needed to increase the authenticity, presence, and immersion of the scene by preparing the materials. Besides, side effects imposed by unrelated factors also needed to be eliminated. So, we completed the following processes:

1. To prevent the materials from bringing the meaning of the marketing content in the real scene, which then poses excessive influences on the subjects, such as the influence caused by matching the subjects' interests, we selected a regular mobile data gifting campaign of China Mobile as the content of the materials. The campaign was not attractive to the subjects, as the subjects had subscribed to a university campus service plan which included a data plan of unlimited mobile data traffic.
2. The materials were processed as follows to prevent factors other than interface layout from affecting the subjects: (1) content on all the materials were made the same (the same mobile data gifting campaign), (2) visual elements other than interface layout (such as pictures, text, and fonts, etc.) were made the same, (3) the materials were made into gray-scale images with the same size, resolution, and aspect ratio, to remove the excessive influence of color and scale.
3. The editing process of the materials should not introduce layouts that do not exist in real-world practice. As one of the promising application fields of our work is the marketing recommendation systems (Xu et al., 2021) which deliver large amounts of marketing content to the customers, we grabbed a large number of mobile marketing campaign interfaces from the mobile recommendation systems on the Internet with a web crawler introduced in one of our previous works (Xiao et al., 2020). We made 30 interface images as the stimulus materials with reference to the layouts of the grabbed interfaces.
4. The number of layout elements was controlled to 9–17 (Mean = 13, SD = 2.4) to prevent over-complexity of the interface in the materials from bringing excessive cognitive overhead.

In addition to the 30 stimulus materials, we made a target stimulus material of a layout framework. It is an abstract interface without marketing content. All materials were placed in a 640x960 pixel rectangular frame which simulates the typical aspect ratio of a mobile phone interface and has a light gray [RGB(242,242,242)] background color. All materials were produced by GIMP 2.10 and exported as smooth raster images.

**Figure 1:**
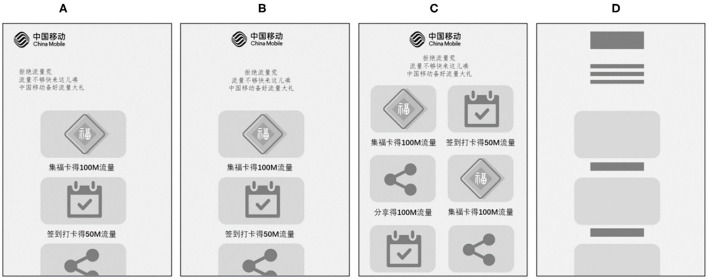
Stimulus material examples. Panels **(A–C)** depict the materials of different layouts while the layouts were designed based on some common mobile marketing campaign interface layouts. Panel **(D)** illustrates the target stimulus which is a toy interface consisting of placeholder geometric figures.

To analyze the impact of the meaning of the marketing content on subjects' aesthetic preferences, we recruited another 20 subjects (aged 22–25, mean = 23.1, SD = 0.9) from the students in the same university. They participated in filling out a 7-point scale of preference for the meaning of the marketing content in the materials (scores from 1 to 7). The scale shows that the mean score was 3.95 (SD = 0.9), indicating that the influence of the meaning of the marketing content in the materials on subjects' aesthetic preferences is neutral. Examples of some of the materials are shown in Figure 1. Figures 1A–C are examples of non-target stimulus, while Figure 1D is the target stimulus material.

**Figure 2:**
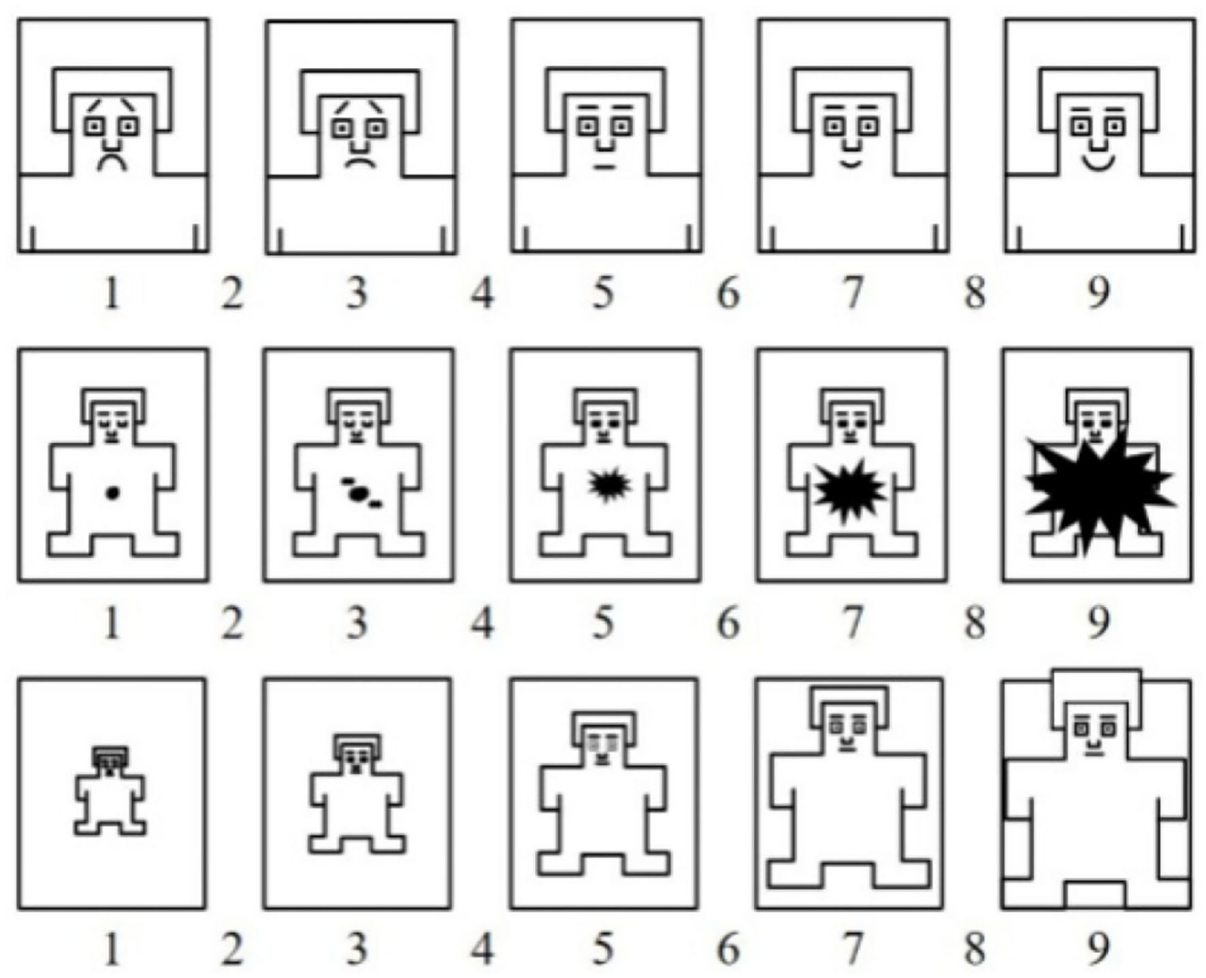
A typical SAM questionnaire.

Under an aimless free-browsing context, users are not supposed to experience severe emotional fluctuations. The materials used in the experiment are not supposed to bring excessive influence on the subjects' emotions. So we tested the materials from the emotional valence (Paradiso et al., 1999) and arousal (Lane et al., 1999) they raised by recruiting another 30 subjects (aged from 22 to 25, mean = 23.5, SD = 1.1) from the local university and asking them to finish a 9-point SAM (Self-Assessment Manikin) questionnaire (Bradley and Lang, 1994; Morris, 1995). SAM is a non-semantic graphical assessment technique used to measure the emotional valence, arousal, and dominance (Yani-de Soriano and Foxall, 2006) of subjects stimulated by different factors. SAM represents the three emotional dimensions of valence, arousal, and dominance by manikins which reduces the need for subjects to rely on texts to state their judgments. The numbers 1-9 are placed below the manikin images and between the gaps of the manikins to represent the corresponding emotional dimension's degree. A typical SAM questionnaire is shown in Figure 2. The first row of manikins represents emotional valence, the second row represents emotional arousal, and the third row represents emotional dominance. The manikins for emotional valence range from a sad/unhappy little man to a happy/smiling little man, as the manikins for emotional arousal range from a close-eyed/sleepy little man to an open-eyed/excited little man. Our stimulus materials are mobile marketing interface images with relatively plain marketing content, making them hard to incur emotional dominance. Thus, we eliminated the emotional dominance part of the SAM questionnaire. The SAM data analysis showed that the mean emotional valence caused by the materials is 4.98 (SD = 0.87), and the mean arousal is 2.99 (SD = 0.81). The results indicate that the materials are neutral in emotional valence and have a low arousal level. Therefore, the materials are suitable for simulating the situation under an aimless free-browsing context.

All stimulus materials were presented on a 19-inch LCD and located right at the center of the screen. The subjects were asked to keep the distance between their eyes and the display around 80 cm. They used a wired optical mouse to respond to the target stimulus during the experiment. The display and mouse were connected to a desktop computer (CPU: i5-4590, Memory: 4GB, OS: Windows 7) which runs the psychology experiment software E-Prime (https://pstnet.com/).

### 2.3. Experiment

**Figure 3:**
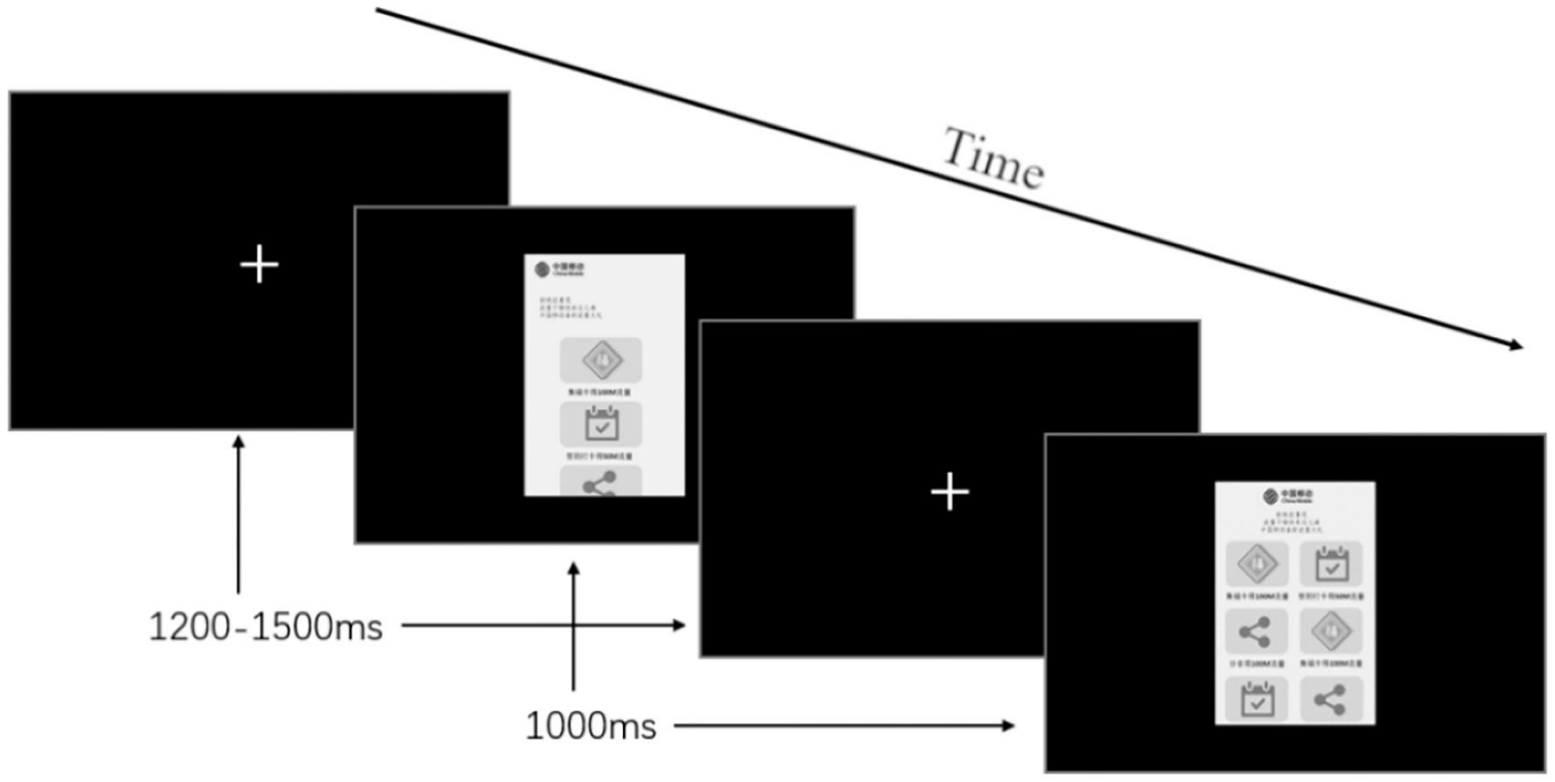
The trial sequence. Two trials with different stimuli. A focus “+” appears at the center of the display for 1,200–1,500 ms randomly and then the stimulus appears for 1,000 ms.

The experiment aims to collect and record the brainwaves evoked by visual stimuli of different mobile marketing interface layouts. The experiment design protects subjects' preferences and emotions from being influenced by layout-unrelated information of the stimuli. The process of the experiment is described as follows:

1. Subject preparation. A subject needs to wash the hair and scalp with electrically neutral shampoo and dry their hair to ensure good electrical conductivity. The subject was then asked to sit in front of the display, adjust the chair's height and the display's angle to make it comfortable while watching the stimuli (the subjects sit 1 m away from the display and the visual is 5.3 degrees) (Ma et al., 2015). The laboratory room is soundproof and electromagnetic radiation-proof with no natural light source. The only artificial light source in the room was dimmed down during the experiment. An electrode cap was placed on the subject's head to collect and record the brainwaves during the experiment. The cap was adjusted to ensure the electrodes were correctly located and did not cause the subject any uncomfortable experience. The conductive paste was then injected into the gaps between the electrodes and the scalp to reduce the electrical impedance to an acceptable level (<5kΩ), indicating the electrodes were in good contact with the scalp.
2. Main experiment process. Before actually presenting the stimuli, an experiment instruction was prompted to the subject. The instruction contained the following information: (1) subjects need to watch the stimuli presented on the screen, and besides the target stimulus, the marketing content on all the other stimuli are the same; (2) when the target stimulus shows up, subjects need to give their responses by clicking the left mouse button as soon as possible; (3) subjects should reduce their head and eyeball movements and keep their eyesight straight. A training session was introduced, which presented the target stimuli for 2 s to help the subject learn how to respond to the target stimuli. The tester left the room after guiding the subject through the instruction, and the experiment was triggered by a right mouse button click issued by the subject. The stimuli were presented in a random sequence on the black backgrounded display [RGB(0,0,0)], making the boundaries of the stimuli clear and identifiable. Each trial started with a frame of a white cross (“+”) at the center of the screen on a black background to refocus the subjects' eye. The frame's presentation duration ranged from 1,200 to 1,500 ms. Following this frame was a frame of a stimulus that lasted for 1,000 ms. The trial sequence is depicted in Figure 3. Each non-target stimuli was presented 50 times to ensure good quality in the “in-place averaging” process during the data analysis. The trials were divided into five groups (each group lasting for around 15 min with 300 trials in it) to mitigate the fatigue and boredom brought by a trial group that was too long. The target stimulus was presented 10 times randomly with the other stimuli in each of the 5 trial groups.
3. Experiment ending. The electrodes and the cap were removed from the subject's head. The subject was then asked to fill in a SAM questionnaire to self-assess the emotional reaction raised by processing the stimuli with the only difference in their layouts. In the study of Li et al. (2015), they asked the subjects to choose 12 Chinese typefaces they liked most, 12 they disliked most, and the rest of the 175 Chinese typefaces were kept as the non-target group. In the study of Ma et al. (Ma et al., 2015), they used 20 top-ranking images of noted-architect-designed architecture, 20 bottom-ranking images of ordinary architecture, and the other 40 were kept as the rest objects. As learnt from their studies, by the end of the experiment, the subjects were presented with 30 interface images used as the stimuli. They were asked to choose, from the stimuli presented in the experiment, five interfaces they liked most and five they disliked most aesthetically, the rest was made up of the “other interfaces.” We've also tested the visual element number difference between the two preference categories. The mean visual element numbers were 13.274 (SD = 2.570) and 12.874 (SD = 2.606) for the liked and disliked stimuli, respectively. The repeated measures ANOVA result showed no significant difference between the visual element numbers of the two categories [*F*_(1,94)_ = 1.004, *p* = 0.319].

### 2.4. EEG Recording and Data Processing

The EEG signal data were recorded with the experimental platform produced by Compumedics Neuroscan (https://compumedicsneuroscan.com/), including a SynAmps 2 signal amplifier (64 channels) and a synthesizer, a Quick-Cap 64 channel electrode cap (complying with the international standard 10-20 system), and a copy of the Curry 8 data acquisition and processing software. The stimuli were presented with a program created by the psychology experiment platform of E-Prime, which generated marks for each stimulus. The EEG signal data captured by the Quick-Cap electrode cap were first amplified by the SynAmps 2 amplifier and then synthesized with the marks generated by the E-Prime program, and finally recorded by the Curry 8 software running on a desktop computer (CPU: i5-4590, Memory: 8GB, OS: Windows 10). The mastoids were selected as the reference for the recording. Two electrodes were attached to the left supraorbital and infraorbital ridges of the left eye, respectively, to record the vertical eye movement. Another two electrodes were attached to the lateral sides of both the left and right eye canthi to record the horizontal eye movement. The skin where the electrodes were attached was treated with a scrub to ensure good skin conductivity. The impedance of all the electrode channels was lowered to below 5KΩ by injecting conductive paste into the gaps between the electrodes and the skin. The sampling rate of the recording was 100 MHz, and a 0.10–100 MHz band-pass filter was applied to the recording. We used an offline approach to process the EEG data, which mainly includes: re-referencing, filtering, artifact removal (eye moving artifacts, muscle movement artifacts, high-frequency artifacts, etc.).

Studies have shown that the ERP components related to aesthetic preference raised by visual stimuli can be recorded from regions across the scalp. Jacobsen et al. studied the aesthetic preference-related ERP components recorded from 25 electrodes located on the brain's frontal, central, parietal, and occipital lobes (Jacobsen and Höfel, 2001). Obermeier et al. studied the aesthetic preference-related ERP components recorded from 36 electrodes across the 4 scalp regions of anterior left, posterior left, anterior right, and posterior right (Obermeier et al., 2016). As learnt from the studies of Li et al. (2015) and Ma et al. (2015), we selected the following electrodes to record and process the EEG signal data: Fz, F1, F2, Cz, C3, C4, Pz, P1, P2, Oz, O1, O2. The electrodes can be divided into frontal-central (Fz, F1, F2, Cz, C3, C4) and parietal-occipital (Pz, P1, P2, Oz, O1, O2). The ERP waveform for each of the subjects was derived from three different situations: (1) the average ERP waveforms of the liked interfaces (5 for each subjects), (2) the average ERP waveform of disliked interfaces (5 for each subjects), (3) the average ERP waveforms of all the rest interfaces which were used as the reference waveforms (20 for each subjects). Each waveform segment lasted for 1,000 ms, with the first 200 ms as the baseline signal. The waveforms which contain signals that exceed ±100 μV were discarded and not used in the averaging process. The exported data were then analyzed by the statistical software Jamovi.

## 3. Result

### 3.1. Behavioral Results

During the experiment, subjects were asked to respond to the target stimulus which demonstrated the attention allocation of the subjects and thus could reveal whether the subjects were paying attention to the visual stimuli. The E-Prime software recorded the keys the subjects pressed to give their response and the reaction time for their actions, so the accuracy could be measured by dividing the number of correct key presses by the total number of the onset of the target stimulus. One of the subjects forgot to take off his smart watch before the experiment, so his data were excluded. The data showed that the average accuracy for the subjects were 0.986 (SD = 0.118), the average reaction time was 536 ms (SD = 83.8). The subjects were asked to fill in a SAM questionnaire (9-point) after the experiment to test the influence of the liked/disliked interfaces (stimulus materials) on the emotional valence and arousal of the subjects. The results show that the average emotional valence for the interfaces liked by the subjects was 5.18 (SD = 0.59) and the average emotional valence for the interfaces disliked by the subjects was 3.93 (SD = 0.61); the average emotional arousal for the interfaces liked by the subjects was 2.24 (SD = 0.51) and the average emotional arousal for the interfaces disliked by the subjects was 3.63 (SD = 0.48). The ANOVA results show that the emotional valence of the liked interfaces significantly surpassed the emotional valence of the disliked interfaces [*F*_(1,18)_ = 206.882, *p* <0.001]; the emotional arousal of the liked interfaces was significantly lower than the emotional valence of the disliked interfaces [*F*_(1,18)_ = 876.113, *p* <0.001].

### 3.2. ERP Results

**Figure 4:**
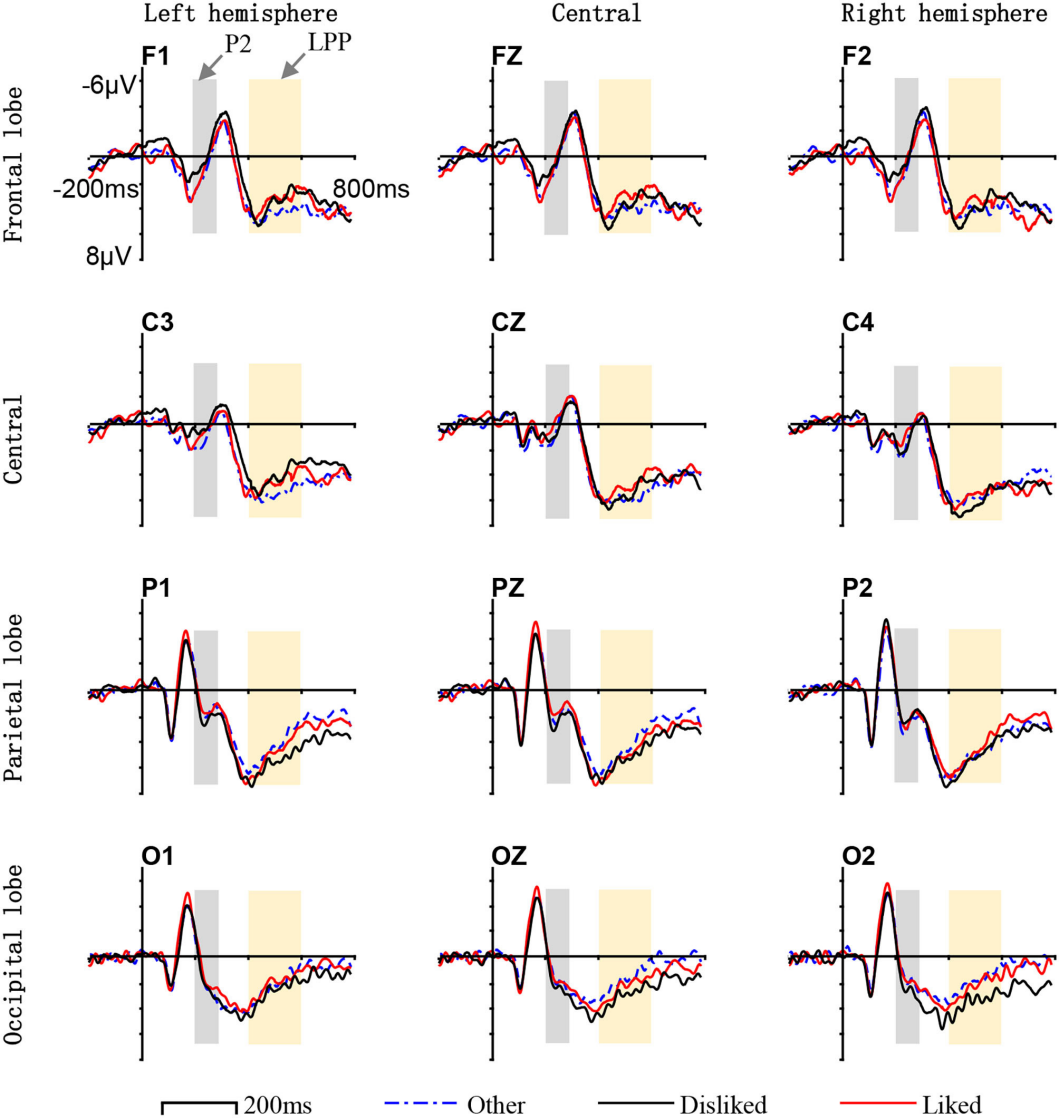
The average ERP waveform for liked/disliked/other interfaces within different scalp areas in different post-stimulus onset time windows. No significant ERP difference for stimuli of different preferences was found in the 150–200 ms time window from the frontal-central or parietal-occipital regions. Significant P2 (marked as gray) differences for stimuli of different preferences from the frontal-central and parietal-occipital areas were found in the 200–300 ms time window. Significant LPP (marked as light yellow) differences for stimuli of different preferences from the frontal-central and parietal-occipital areas were found in the 400–600 ms time window. The x-axis indicates the time and the y-axis indicates the voltage.

**Figure 5:**
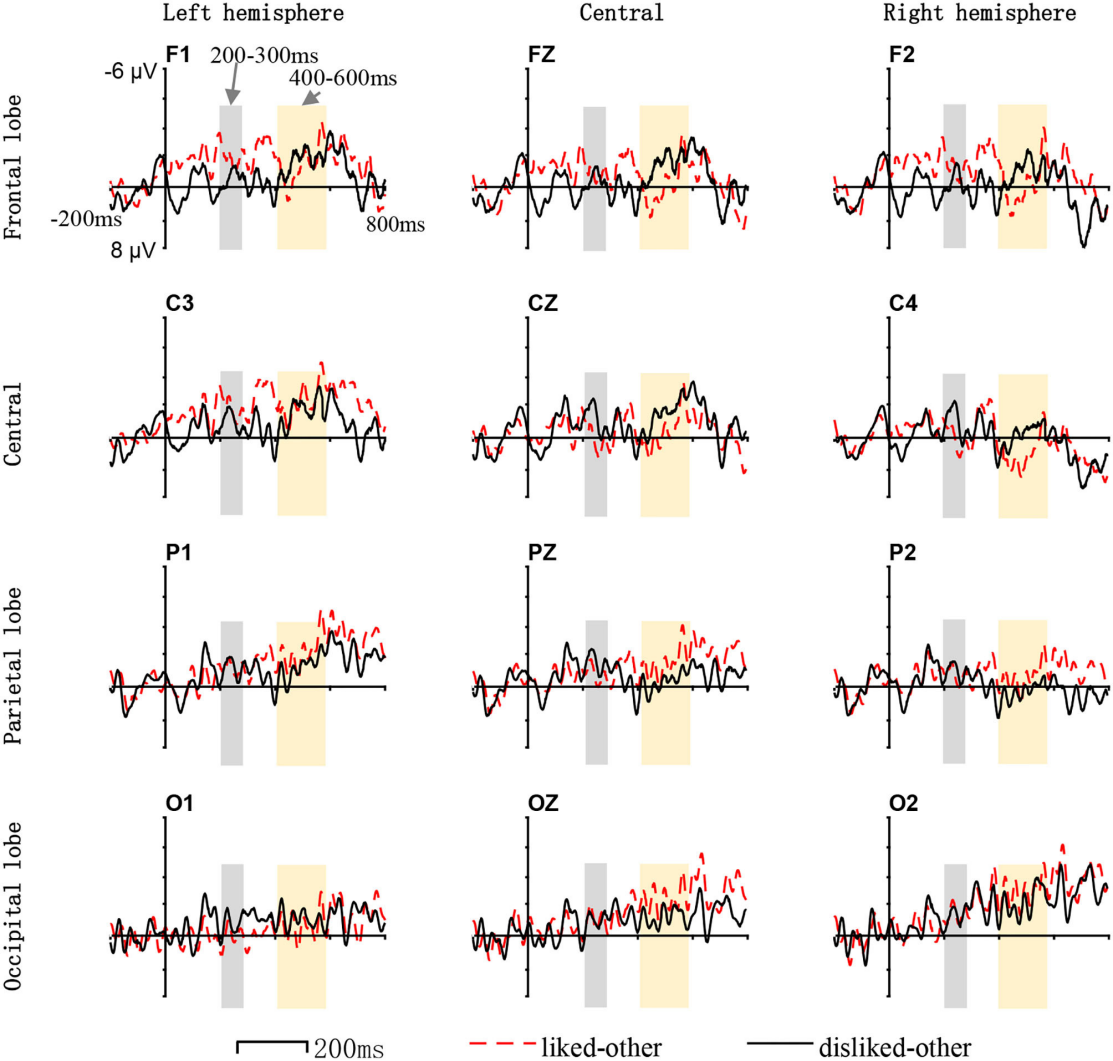
Difference ERP waveforms of comparing waveforms of liked/disliked/other stimuli. The waveforms were constructed by using (1) the grand average ERP waveforms for the liked interfaces against that of the other interfaces and (2) the grand average ERP waveforms for the disliked interfaces against that of the other interfaces, for each electrode site. The x-axis indicates the time and the y-axis indicates the voltage. The gray mark indicates the 200–300 ms time window, the light yellow mark indicates the 400–600 ms time window.

**Figure 6:**
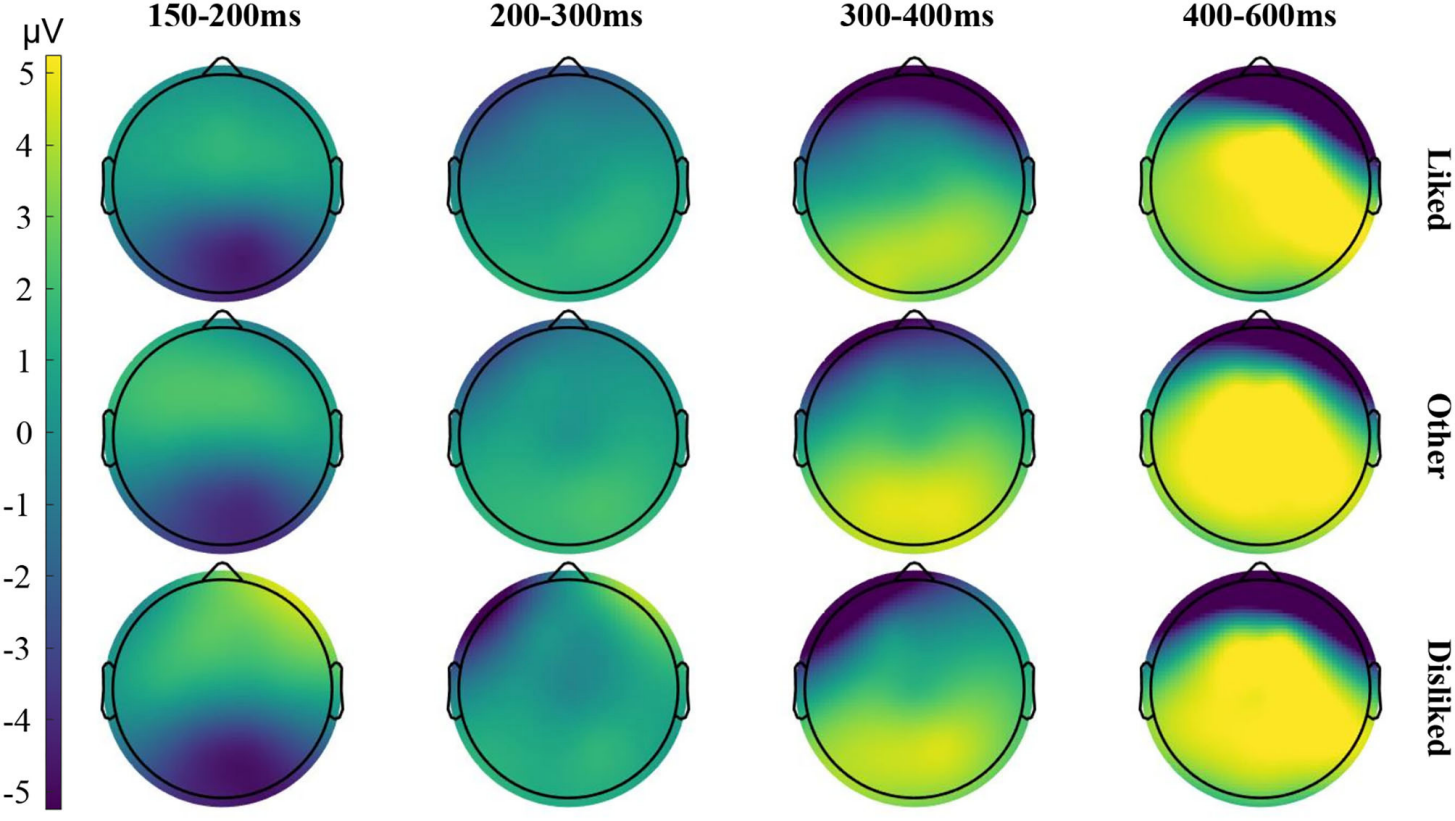
Brain voltage maps for liked/disliked/other stimuli across different time window. The maps were constructed based on the grand average ERP waveforms recorded under each condition.

The ERP waveforms derived from 19 subjects (one subject's data were excluded) and their choices of liked/disliked interfaces (the stimulus images with the only difference in layout) were analyzed. The average ERP amplitudes in different post-stimulus onset time windows (150–200, 200–300, 300–400, and 400–600 ms), measured from different scalp areas (frontal-central area and parietal-occipital area), are listed in Table 1. The average ERP waveforms for liked, disliked, and other interfaces (non-target stimulus other than the liked and disliked interfaces) respectively are shown in Figure 4. The differences in ERP waveforms constructed by comparing the waveforms of liked/disliked/other stimuli are shown in Figure 5. The brain voltage maps for liked/disliked/other stimuli across the four post-stimulus onset time windows are shown in Figure 6.

**Table 1:** Average ERP amplitudes within each scalp area in each post-stimulus onset time window (standard deviations are in parentheses).

	**Frontal-central**			**Parietal-occipital**		
**Time window (ms)**	**Left**	**Central**	**Right**	**Left**	**Central**	**Right**
150–200	1.96 (3.46)	1.76 (3.60)	1.82 (3.39)	−1.94 (4.19)	−2.40 (4.28)	−2.69 (4.13)
200–300	1.86 (3.84)	1.57 (3.75)	2.29 (3.63)	1.63 (3.78)	1.89 (3.83)	2.09 (3.83)
300–400	2.00 (4.75)	1.85 (4.39)	2.58 (4.44)	4.30 (3.96)	4.22 (3.90)	4.26 (3.78)
400–600	4.58(4.45)	4.86 (4.48)	5.02 (4.51)	4.12 (3.58)	4.22 (3.48)	4.21 (3.27)

The ERP data are statistically analyzed by repeated-measures ANOVAs on each of the four post-stimulus onset time windows. The factors are preference (liked, disliked, and other) and laterality (left hemisphere, central, right hemisphere). The sphericity assumption of the ANOVAs is checked and tested by Mauchly's sphericity test. The results are corrected by Greenhouse-Geisser epsilon.

The results for the four time windows are listed below.

1. The 150–200 ms post-stimulus onset time window: ERP data in this time window are related to the ERP component of P1 from the frontal-central area and N1 from the parietal-occipital area. In the frontal-central area (P1), no significant main effect is found for preference (like, dislike, and other) [*F*_(2,36)_ = 0.150, *p* = 0.700] and the interaction between preference and laterality (left hemisphere, central, right hemisphere) [*F*_(2,72)_ = 0.185, *p* = 0.832], but a significant main effect is found for laterality [*F*_(2,36)_ = 35.246, *p* <0.01]. In the parietal-occipital area (N1), no significant main effect is found for preference [*F*_(2,36)_ = 0.430, *p* = 0.652] and the interaction between preference and laterality [*F*_(2,72)_ = 0.215, *p* = 0.807)], but a significant main effect is found for laterality [*F*_(2,36)_ = 34.046, *p* <0.01].
2. The 200–300 ms post-stimulus onset time window: ERP data in this time window are related to the ERP component of P2 from both the frontal-central area and the parietal-occipital area. In the frontal-central area (P2), a significant main effect is found for preference [*F*_(2,36)_ = 30.637, *p* <0.01]. No significant main effect is found for laterality [*F*_(2,36)_ = 0.543, *p* = 0.465] or the interaction between preference and laterality [*F*_(2,72)_ = 0.994, *p* = 0.374]. Meanwhile, the difference between the preference factor levels of liked and disliked is insignificant [*F*_(1,18)_ = 1.067, *p* = 0.307], the difference between liked and other is also insignificant [*F*_(1,18)_ = 0.527, *p* = 0.206], but the difference between disliked and other is significant [*F*_(1,18)_ = 4.549, *p* = 0.038]. In the parietal-occipital area (P2), no significant main effect is found for preference [*F*_(2,36)_ = 0.275, *p* = 0.760], laterality [*F*_(2,36)_ = 0.506, *p* = 0.604], and the interaction between preference and laterality [*F*_(2,72)_ = 1.411, *p* = 0.241].
3. The 300–400 ms post-stimulus onset time window: ERP data in this time window are related to the ERP component of N2 from both the frontal-central area and the parietal-occipital area. In the frontal-central area (N2), no significant main effect is found for preference [*F*_(2,36)_ = 2.025, *p* = 0.137], laterality [*F*_(2,36)_ = 0.167, *p* = 0.684], and the interaction between preference and laterality [*F*_(2,72)_ = 0.183, *p* = 0.833]. In the parietal-occipital area (N2), no significant main effect is found for preference [*F*_(2,36)_ = 2.323, *p* = 0.103] and the interaction between preference and laterality [*F*_(2,72)_ = 0.351, *p* = 0.705], but a significant main effect is found for laterality [*F*_(2,36)_ = 31.048, *p* <0.01].
4. The 400–600 ms post-stimulus onset time window: ERP data in this time window are related to the ERP component of LPP from both the frontal-central area and the parietal-occipital area. In the frontal-central area (LPP), a significant main effect is found for preference [*F*_(2,36)_ = 6.745, *p* <0.01], but no significant main effect is found for laterality [*F*_(2,36)_ = 0.683, *p* = 0.413] and the interaction between preference and laterality [*F*_(2,72)_ = 0.783, *p* = 0.460]. In this area, the difference between the preference factor levels of liked and disliked is significant [*F*_(1,18)_ = 16.718, *p* <0.01], as well as the difference between liked and other [*F*_(1,18)_ = 21.810, *p* <0.01] and the difference between disliked and other [*F*_(1,18)_ = 34.841, *p* <0.01]. In the parietal-occipital area (LPP), a significant main effect is found for preference [*F*_(2,36)_ = 26.534, *p* <0.01], a significant main effect is found for laterality [*F*_(2,36)_ = 4.23, *p* = 0.017], but no significant main effect is found for the interaction between preference and laterality [*F*_(2,72)_ = 0.238, *p* = 0.788]. In this area, the difference between the preference factor levels of liked and disliked is insignificant [*F*_(1,18)_ = 3.058, *p* = 0.087], as well as the difference between liked and other [*F*_(1,18)_ = 1.718, *p* = 0.196], but the difference between disliked and other is significant [*F*_(1,18)_ = 24.690, *p* <0.001].

## 4. Discussion

This study aims to test whether the interface layout can cause human implicit aesthetic preference or not under the free-browsing context of mobile marketing which acts as evidence for the mechanism of interface layout affecting the human aesthetic experience. By presenting the subjects with a series of mobile marketing interfaces of the same marketing content with the only difference in layout, we recorded and analyzed the behavioral data of the subjects during the experiment and the brain activity signals evoked by these visual stimuli, and analyzed the data with the theories and techniques of event-related potentials.

During the processing of visual stimuli and generation of aesthetic preference judgments, the brain launches information processing and emotion processing in parallel. Moreover, though some studies using the PPM theory argue that emotion drives decision Xiang et al. (2021), the emotion processing reveals the process of aesthetic preference processing. Vartanian et al. suggested that for people without aesthetic-related training, the emotion pathways are activated for aesthetic preference activities Vartanian and Goel (2004). Nadal et al. revealed the connection of emotional processing and aesthetic preference using neuroimaging techniques (Nadal et al., 2008). The framework for the aesthetic preference of human brains processing visual stimuli (Chatterjee, 2003) suggested a solid support for the connection of emotional processing and aesthetic preference processing. The framework treats the processing of aesthetic preference as a 3-stage process. In the first stage, the visual circuitry processes the visual stimuli at the early stage, extracts information from the visual stimuli. In the second stage, parts of the extracted information are segmented, and parts are grouped to form a coherent visual expression. In the third stage, some features of the stimuli are selected for further investigation, which triggers emotional processing. The framework and the development of cognitive neuroscience promote the development of the scientific field of neuroaesthetics (Skov and Vartanian, 2009; Chatterjee, 2011; Chatterjee and Vartanian, 2014).

The behavioral results suggest that the subjects performed well during the experiment and under the free-browsing context the layout differences influence the subjects' emotional appraisals. The behavioral data collected during the experiment by asking the subjects to respond to the target stimulus with left mouse button clicks and the data of the SAM questionnaire have also supported our hypothesis. Many factors such as culture, gender, age, education, growth environment, etc., affect human aesthetic preference (Sevenant and Antrop, 2010; Street et al., 2016). These factors generate individual aesthetic preference differences. In our study, we treat the subjects as a whole while investigating whether human beings make rapid aesthetic preferences to the layouts of mobile marketing interfaces. The behavioral data suggest a 98.6% accuracy of responding to the target stimulus, indicating the subjects were energetic and focused on the task during the experiment. This design built the context of free-browsing into the experiment and made the observation of the non-target stimuli (mobile marketing interface images different in layouts) an implicit appraisal. Meanwhile, after the experiment, the subjects made the explicit appraisal of the stimuli by choosing five interfaces they liked and five they disliked. Combining these choices and the SAM questionnaires the subjects had finished, we find significant differences in emotional valence and arousal of the liked and disliked interfaces. This suggests that different mobile marketing interface layouts have different emotional valence and arousal in the explicit aesthetic appraisal, affecting subjects' aesthetic preferences. Another interesting thing is that, among the 30 stimuli, none were significantly favored by the subjects, which indicates that all the layouts used in the experiment have their audience. We think the reason for this phenomenon lies in material preparation. Studies have shown that novelty in visual stimuli is associated with emotion fluctuations (Yanagisawa et al., 2019). To prevent highly novel visual stimuli from bringing excessive emotion fluctuations to the subjects, we designed the layouts used in our experiment by avoiding introducing layouts that do not exist in real-world practice. Therefore, the interface layouts were designed according to the interface layout designs of mobile marketing advertisements grabbed from marketing recommendation systems. Each of the layouts has been used by the marketers in their daily marketing practice, and it is reasonable to conclude that these interface layout designs all have their own audience due to the subjects' individual differences in preference for the interface layout.

The ERP results suggest that human beings do make rapid implicit aesthetic preferences for the layout of interfaces under the free-browsing context in mobile marketing. The suggestion is supported by the differences in the P2 and LPP components which are sensitive to the emotional valence in perceiving visual stimuli of different aesthetic preferences (liked/disliked/other).

In the 200–300 ms post-stimulus onset time window, by comparing the ERP component of P2 from the frontal-central area of the scalp, the brain activities evoked by the disliked interfaces and the other interfaces are significantly different. In the 400–600 ms time window, by comparing the ERP component of LPP from both the frontal-central and parietal-occipital areas of the scalp, the brain activities evoked by the liked, disliked, and other interfaces are significantly different. In comparing P1, N1 in the 150–200 ms time window, and N2 in the 300–400 ms time window, no significant difference can be found between brain activities evoked by liked, disliked, and other stimuli. So, we can deduce from the result that significantly different brain activities are mainly evoked by disliked interfaces.

In the 200–400 ms post-stimulus onset time window (200–300 and 300–400 ms), the P2 and N2 are the main ERP components related to the perceptual aspect of visual appraisal according to cognitive neuroscience (Mangun, 1995; Wijers, 1995; Carretié et al., 1997; Höfel and Jacobsen, 2007; de Tommaso et al., 2008). Studies have shown that when compared with neutral visual stimuli, both positive and negative visual stimuli cause an increment in the amplitudes of the P2/N2 components (Herbert et al., 2006; Scott et al., 2009). These studies have confirmed that the P2 and N2 ERP components are related to the aesthetic preference associated with the emotional valence in perceiving visual stimuli. We have found significant differences for the P2 components evoked by disliked and other interfaces from the frontal-central area of the scalp. Di Russo et al. showed that the P2 component in the 200–300 ms time window reflects the early visual discrimination process of the visual stimuli (Di Russo et al., 2006). Therefore, our findings confirm that in the 200–300 ms time window, the observed differences reflect the rapid identification and discrimination of interface layouts in the early stages of the human cognitive process.

In the 400–600 ms post-stimulus onset time window, we mainly focus on the LPP ERP component. In our work, the statistical data in this time window reveal that the LPP waveforms of the liked, disliked, and other interfaces are significantly different. The average LPP amplitude for disliked interfaces is greater than that of the liked or other interfaces. Many cognitive neuroscience studies have confirmed that the LPP component in the 400–600 ms time windows is substantially sensitive to the emotional valence of visual stimuli (Cacioppo and Berntson, 1994; Cuthbert et al., 2000; Olofsson et al., 2008; Handy et al., 2010; Mickleborough et al., 2014). Some studies also reveal that LPP is stronger in reacting to emotional stimuli than neutral ones (Cuthbert et al., 2000; Schupp et al., 2000; Olofsson et al., 2008; Pastor et al., 2008; Foti et al., 2009; Hajcak et al., 2010). The experiment design of our study makes layout the main factor that causes the ERP difference for liked, disliked, and other interfaces. Based on these studies, our finding contributes to the evidence that interface layouts have emotional valence that influences human preference processing. The layouts of disliked interfaces have stronger emotional valence.

According to Chatterjee's study (Chatterjee, 2003), incorporating non-perceptual processes (such as emotions) contributes to the main difference between aesthetic preference and other cognitive processes in dealing with visual stimuli. By combining this finding and the results of the SAM questionnaire to interpret the experimental results, we conclude that the layout of mobile marketing interfaces impacts the human cognitive process. The two significant differences in the experimental results (200–400 ms: P2 difference from the frontal-central area, 400–600 ms: LPP peaks at the central-parietal area and shows significant differences across the scalp) suggest that the layout affects the human cognitive process, which further influences decision-making. Our finding supports the fact that the layout can be seen as a kind of emotional stimulus (the same as logos or geometric figures Bargh and Ferguson, 2000; Handy et al., 2010), which causes human aesthetic preference.

Visual attention contributes to the influence of interface layout on aesthetic preference together with emotional processing. The experiment design mimicking the free-browsing context in mobile marketing has side effect of manipulating the subjects' visual attention. Moreover, from the ERP results we find that the differences in the ERP components are mainly caused by the disliked interface layouts. These influences can be expressed by the differences in the ERP components of P2 and LPP which are also closely related to attention level.

Though the ERP results reveal a significant difference in brain activities between liked and other interfaces from the frontal-central area in the 400–600 ms time window. We still believe the aesthetic cognition for the visual stimuli of interfaces is influenced by interface layouts and mainly by disliked interface layouts. Because the ERP components evoked by the liked interface layouts in the other time windows as well as the ERP component evoked by the liked interface layouts in the 400–600 ms from the parietal-occipital area show no significant difference to those evoked by other interfaces. The mouse click task in the experiment yields high accuracy, and the subsequent questionnaire also shows good performance (no hesitation and long pauses in memory recall). Therefore, the insignificance of the liked and other interfaces' ERP difference might result from the experiment task and the stimulus material preparation. The subjects were asked to focus on the target stimuli and make responses. Many studies have shown that the amplitude of LPP falls significantly as the attention level drops (Fox, 1994; Liberzon et al., 2000; Bishop et al., 2004, 2007; Pessoa et al., 2005; Mothes-Lasch et al., 2013). The studies on working memory have also suggested that working memory load decreases the amplitude of LPP (MacNamara et al., 2011, 2018; MacNamara and Proudfit, 2014). These findings indicate that attention level and working memory load affect LPP.

In our work, subjects were asked to focus on the target stimulus, resulting in subjects allocating most of their attention to the target stimulus. Thus, little attention was allocated to the non-target stimuli, and the subjects' working memory loads were increased. Therefore, no significance was observed for the liked and other interfaces' ERP difference. Meanwhile, our subjects had little knowledge of art, design, or aesthetics, making them unaware of the aesthetic aspect of the non-target stimuli in the experimental context. Also, the materials were prepared with low emotional arousal to avoid bringing too many emotional fluctuations. This adds to the unawareness of the subjects to the subtle differences in the interface layouts. Stimuli with high emotional arousal lead to high LPP amplitudes (Schupp et al., 2003), so that LPP can detect a continuous increase in attention caused by emotional stimuli (Hajcak et al., 2006). Ferrari et al. suggest that LPP amplitude reflects attention allocation (Ferrari et al., 2011). Leite et al. support the findings of Ferrari et al. and confirm that high arousal valid emotional stimuli cause significantly more increments in LPP than low arousal valid emotional stimuli. The increments in LPP reflect more attention allocation (Leite et al., 2012). These studies have proved that the changes in LPP are closely related to attention and arousal, which offer a theoretical basis for our work. The LPP amplitude caused by the disliked interfaces is greater than those caused by the liked and other interfaces. This can result from an attention level rise caused by the disliked interfaces that can raise more emotional arousal than the liked ones.

Aside from LPP, we also find a significant difference in brain activities (P2) evoked by disliked and other interfaces during the 200–300 ms time window, which can be explained by attention level. De Tommaso et al. suggest that visual stimuli, which cause increases in arousal, cause increments in attention level even without explicit aesthetic appraisal (de Tommaso et al., 2008). Carreti et al. reveal that after the initial perception of the visual stimuli, the ERP component of P2 reflects the selection attention of the visual stimuli (Carretié et al., 2004). The visual stimuli random repetition of the experiment design influences the selection attention of the subjects. Subjects get more familiar with the stimuli as they continuously appear to them, leading to the result that stimuli with lower arousal get lower attention levels. The familiarity process also explains the high accuracy in the mouse click task and the good performance in the subsequent questionnaire.

In addition to layout evoking aesthetic preference, our work also assumes that the aesthetic preference of human beings to layout is implicit. Some studies suggest that when browsing passively, people do implicit appraisals to visual stimuli (Handy et al., 2010; Wang et al., 2012). In our work, the experiment was designed to reflect the situation of free-browsing. Subjects were instructed to focus on detecting the target stimulus and making responses. In this experiment process, no explicit aesthetic appraisals were required for the non-target stimuli. The ERP component of N1 is suggested to be closely related to conscious attention strategies (Mangun, 1995; Handy and Mangun, 2000). We found no significant N1 difference in the 150-200 ms time window, which supports the fact that the subjects did not do explicit aesthetic appraisals for the visual stimuli in our experiment. The results also show that the layouts of disliked interfaces produce the majority of the differences. Thus, our work shows that people have implicit aesthetic preferences for the layouts of mobile marketing interfaces.

## 5. Conclusion

Users interact with mobile marketing systems by their interfaces, whether the design factors of interfaces can interact with user preferences and better attract users under free-browsing context has always been a primary focus of businesses, designers, and researchers. Neuroaesthetics offers a prominent perspective to study the problem. In this paper, we take the cognitive neuroscience approach of ERP methodology to study whether mobile marketing interface layout can trigger human aesthetic preference. The brain activity data are collected by a designed experiment that records subjects' brainwaves as mobile marketing interface images with identical marketing content and different layouts are presented together with a target stimulus to the subjects. The ERP data extracted from four post-stimulus onset time windows as well as the behavioral data are analyzed. The results support our hypothesis by showing that the differences in the cognitive processing of the three kinds of visual stimuli (the liked, disliked, and other interfaces) are reflected by the differences in the corresponding ERP waveforms. The results also suggest that although users tend to skim through a large number of mobile marketing contents in different interface designs under a free-browsing context, interface layouts that raise high emotional arousal are still able to attract more attention and induce implicit aesthetic preference. The findings of this work support the hypothesis that humans do have rapid implicit aesthetic preferences for mobile marketing interface layouts. The findings of our study are critical to understand the mechanism of how the interface layout affects the human aesthetic experience and can benefit the mobile marketers by providing a deeper understanding of their marketing interface designs, especially during the COVID-19 pandemic.

## Data Availability Statement

The original contributions presented in the study are included in the article/supplementary material, further inquiries can be directed to the corresponding author/s.

## Ethics Statement

The studies involving human participants were reviewed and approved by the Ethics Committee of Zhejiang Gongshang University. The patients/participants provided their written informed consent to participate in this study.

## Author Contributions

LX and SW developed the study concept. CX contributed to the design. SW collected the data and was supported by LX. SW and CX analyzed the data. LX provided the analysis tool. SW wrote the first draft of the manuscript. LX acquired the funding. All authors helped to edit and revise the manuscript and approved the final submitted version of the manuscript.

## Funding

This paper was supported by the Key Research Institute (KRI) - Modern Business Research Center of Zhejiang Gongshang University and the Special fund for basic operating expenses of Zhejiang Province-owned colleges and universities (No. 2021SM02YB) and the National Social Science Fund of China (No. 19BGL098).

## Conflict of Interest

The authors declare that the research was conducted in the absence of any commercial or financial relationships that could be construed as a potential conflict of interest.

## Publisher's Note

All claims expressed in this article are solely those of the authors and do not necessarily represent those of their affiliated organizations, or those of the publisher, the editors and the reviewers. Any product that may be evaluated in this article, or claim that may be made by its manufacturer, is not guaranteed or endorsed by the publisher.
